# Endogenous PCSK9 may influence circulating CD45^neg^/CD34^bright^ and CD45^neg^/CD34^bright^/CD146^neg^ cells in patients with type 2 diabetes mellitus

**DOI:** 10.1038/s41598-021-88941-x

**Published:** 2021-05-06

**Authors:** Romina Tripaldi, Paola Lanuti, Paola Giustina Simeone, Rossella Liani, Giuseppina Bologna, Sonia Ciotti, Pasquale Simeone, Augusto Di Castelnuovo, Marco Marchisio, Francesco Cipollone, Francesca Santilli

**Affiliations:** 1 Department of Medicine and Aging Sciences, Center for Advanced Studies and Technology (CAST), Via Luigi Polacchi, Chieti, Italy; 2 Mediterranea Cardiocentro, Napoli, Italy

**Keywords:** Endocrine system and metabolic diseases, Biological techniques, Molecular biology, Biomarkers, Endocrinology

## Abstract

Protease proprotein convertase subtilisin/kexin type 9 (PCSK9) is a regulator of LDL cholesterol clearance and has been associated with cardiovascular risk. PCSK9 inhibitors increase in vivo circulating endothelial progenitor cells (EPCs), a subtype of immature cells involved in ongoing endothelial repair. We hypothesized that the effect of PCSK9 on vascular homeostasis may be mediated by EPCs in patients with or without type 2 diabetes mellitus (T2DM). Eighty-two patients (45 with, 37 without T2DM) at high cardiovascular risk were enrolled in this observational study. Statin treatment was associated with higher circulating levels of PCSK9 in patients with and without T2DM (p < 0.001 and p = 0.036) and with reduced CD45^neg^/CD34^bright^ (total EPC compartment) (p = 0.016) and CD45^neg^/CD34^bright^/CD146^neg^ (early EPC) (p = 0.040) only among patients with T2DM. In the whole group of patients, statin treatment was the only independent predictor of low number of CD45^neg^/CD34^bright^ (β = − 0.230; p = 0.038, adjusted R^2^ = 0.041). Among T2DM patients, PCSK9 circulating levels were inversely related and predicted both the number of CD45^neg^/CD34^bright^ (β = − 0.438; p = 0.003, adjusted R^2^ = 0.173), and CD45^neg^/CD34^bright^/CD146^neg^ (β = − 0.458; p = 0.002, adjusted R^2^ = 0.191) independently of age, gender, BMI and statin treatment. In high-risk T2DM patients, high endogenous levels of PCSK9 may have a detrimental effect on EPCs by reducing the endothelial repair and worsening the progression of atherothrombosis.

## Introduction

Diabetes mellitus is associated with accelerated atherogenesis of the coronary, peripheral, and cerebrovascular districts and with a twofold higher risk of atherothrombotic events, as compared to subjects without diabetes, despite extensive treatment with state-of-the art preventive therapies, including statins, anti-hypertensives, antiplatelet agents. There is urgent need to identify novel strategies tackling the residual risk of cardiovascular events.

Proprotein convertase subtilisin–kexin type 9 (PCSK9) is a secreted serine protease that binds to the extracellular domain of the low density lipoprotein (LDL) receptor and targets the LDL receptor to the lysosomal compartment for degradation^1^. PCSK9 is secreted mainly by the liver and is found in 1 in every 500 LDL particles in the circulation^2^. Mutations in PCSK9 have been associated with autosomal dominant hypercholesterolemia^3^.

Large trials have reported that PCSK9 inhibitors reduce LDL cholesterol and the risk of major cardiovascular (CV) events^4^, indicating PCKS9 as a novel therapeutic target, and establishing the indication to PCSK9 inhibitor treatment for patients who cannot reach the target LDL-C level when taking the maximum-tolerated dose of a statin^5^. A subsequent metanalysis revealed that the effects of PCSK9 inhibitors are comparable in patients with and without type 2 diabetes mellitus (T2DM)^6^.

Although the effect of PCSK9 inhibitors in cardiovascular prevention has been ascribed primarily to the strong LDL-cholesterol reducing action, additional mechanisms have been described such as the modulation of platelet function and blood coagulation^7^ and anti-atherosclerotic pleiotropic effects^8^. The administration of anti-PCSK9 antibody inhibits markers of inflammation and atherosclerosis and interestingly, leads to an increase in circulating endothelial progenitor cells (EPCs) and angiogenic cells in a mouse model^9^ and recently in patients with cardiovascular disease^10^.

This mechanism may be particularly important since EPCs have been defined as “biomarkers” of endothelial function^11^, have a role in ongoing endothelial repair^12^ and their reduction has been linked to CV outcomes^13^. Indeed, after adjustment for relevant variables, increased levels of EPCs were associated with a reduced risk of death from CV causes, first major CV event, revascularization, and hospitalization^14^.

EPCs are damaged in disease. In patients with T2DM, EPCs exhibit impaired proliferation, adhesion, and incorporation into vascular structures^15^ and in patients at high risk for CV events EPCs show higher senescence. This may lead to EPCs depletion, thus impairing the repair of vessel walls and favouring the progression of CV disease^16^.

CD34 is a common marker for diverse progenitors, including hematopoietic stem and progenitor cells, vascular endothelial progenitors^17^, cardiomyocytes and smooth muscle cells^18^. Human CD34^pos^ cells are provided with vascular regenerative capacity and proangiogenic potential in vivo^19^, and their depletion is now considered a significant contributor to the impaired coronary endothelial dysfunction^20^ and altered cardiovascular homeostasis in diabetes. A useful marker to identify non hematopoietic stem and progenitor cells is the absence of the hematopoietic antigen CD45. The robust proliferative potential and endothelial colony forming capacity of CD45^neg^/CD34^pos^ was confirmed in vitro^21^. During the clinical onset of T2DM, a progressive reduction of CD34pos progenitor cells has been observed^22^.

However, CD34 is also expressed on mature circulating endothelial cells (CECs), and thus additional and appropriate antigens are required to discriminate between CECs and EPCs. CD146 is a marker that may further help the characterization. Shim et al. identified two EPC subpopulations based on CD146, which is not expressed on early outgrowth EPCs (stemming from the bone marrow), whereas late outgrowth EPCs do express CD146^23^. While the phenotype of mature CECs (CD45^neg^/CD34^bright^/CD146^pos^) is well established^24,25^, the antigen pattern identifying EPCs has not been clarified yet. In any case, the circulating population of CD45^neg^/CD34^bright^/CD146^neg^ cells results enriched in the earlier EPCs^23,26^. Another subpopulation of CD34^pos^ cells enriched in EPCs co-expresses CD309 or kinase insert domain receptor (KDR) or vascular endothelial growth factor receptor-2 (VEGFR2)^27^. Several authors have defined late circulating EPCs as CD34^pos^CD309^pos^ cells, proving that cells characterized by this phenotype are able to stimulate angiogenesis in vivo^27^. Confirming these findings, Sandhu et al.^28^ also demonstrated that, in parallel with the progression toward the endothelial cell maturation lineage, CD309 and other endothelial markers increase their expression levels. Taking into account these results we may define the CD45^neg^/CD34^bright^/CD146^neg^ subset as the early EPC compartment, while the expression of CD309 within the CD45^neg^/CD34^bright^ may be associated with the late EPC subtype.

We hypothesized that the effect of PCSK9 on vascular homeostasis may be mediated by EPCs. Thus, the aim of this study was to evaluate the relationship between circulating levels of PCSK9 and circulating EPCs as assessed by the number of CD45^neg^/CD34^bright^ (total EPC compartment), CD45^neg^/CD34^bright^/CD146^neg^ (early EPC) and CD45^neg^/CD34^bright^/CD309^pos^ (late EPC) and on CEC, as reflected by CD45^neg^/CD34^bright^/CD146^pos^, and their mutual relationship, in patients at risk of CV disease, with or without T2DM.

## Results

### Baseline characteristics

Eighty-two patients were enrolled, 45 with and 37 without T2DM. Baseline characteristics of patients are reported in Table 1.

**Table 1:** Baseline characteristics.

Variable	No T2DM (n.37)	T2DM (n.45)	p-value^a^
Gender (male), n (%)	22 (59.4)	25 (55.5)	0.448
Age (years)	66 (58–75)	68 (65–71)	0.417
Weight (Kg)	77 (68–91)	81 (74–92)	0.323
BMI (kg/m^2^)	28.2 (25.1–32.3)	30.1 (27.4–34.4)	0.079
Systolic BP (mmHg)	140.0 (127.5–158.0)	144.0 (135.5–150.5)	0.566
Diastolic BP (mmHg)	80.0 (70.5–87.0)	77.0 (70.0–83.0)	0.141
Hypertension, n (%)	33 (89.2)	39 (86.6)	0.500
Fasting plasma glucose (mmol/L)	5.2 (4.7–5.6)	6.7 (5.7–7.4)	< 0.001
HbA1c (mmol/mol)	39.0 (34.0–42.0)	51.0 (43.0–56.0)	< 0.001
Total cholesterol (mmol/L)	5.0 (4.0–5.6)	4.6 (3.9–5.2)	0.213
LDL cholesterol (mmol/L)	2.8 (2.1–3.4)	2.4 (2.0–3.0)	0.198
HDL cholesterol (mmol/L)	1.3 (1.1–1.5)	1.3 (1.1–1.4)	0.588
Triglycerides (mmol/L)	1.3 (1.0–1.7)	1.4 (1.1–1.9)	0.386
Creatinine (μmol/L)	70.4 (61.6–88.0)	70.4 (61.6–79.2)	0.654
eGFR (ml/min)	89.3 (72.0–100.0)	88.0 (78.0–95.5)	0.889
hs-C-reactive protein (nmol/L)	23.8 (7.6–46.7)	29.5 (10.5–63.8)	0.404
AST (U/L)	23.0 (20.5–28.5)	25.0 (20.0–31.0)	0.712
ALT (U/L)	27.0 (23.5–32.0)	30.0 (21.0–40.5)	0.393
Uric Acid (μmol/L)	351.0 (273.6–422.3)	333.1 (276.6–422.3)	0.456
CVD, n (%)	15 (40.5)	16 (35.5)	0.407
Diabetes duration	–	6 (2.5–10)	–
Stable CAD, n (%)	1 (2.7)	5 (11.1)	0.152
Previous MI, or revascularization, n (%)	6 (16.2)	6 (13.6)	0.493
Previous TIA/stroke, o revascularization, n (%)	6 (16.2)	2 (4.4)	0.079
PAD, n (%)	0 (0)	3 (6.6)	0.160
Microvascular disease, n (%)	0 (0)	1 (2.2)	0.549
Retinopathy, n (%)	0 (0)	1 (2.2)	0.549
Chronic renal disease, n (%)	1 (2.7)	0 (0)	0.451
**Medications**			
Metformin, n (%)	1 (2.7)	27 (60)	< 0.001
Sulfonylureas, n (%)	0 (0)	2 (4.4)	0.298
Glinides, n (%)	0 (0)	4 (8.8)	0.085
PPAR-gamma, n (%)	0 (0)	7 (15)	0.012
GLP1RA, n (%)	0 (0)	1 (2.2)	0.549
DPP-IVi, n (%)	0 (0)	1 (2.2)	0.549
Acarbose, n (%)	0 (0)	1 (2.2)	0.549
Insulin, n (%)	0 (0)	0 (0)	1.000
SGLT2i, n (%)	0 (0)	0 (0)	1.000
ACE-I, n (%)	11 (29.7)	16 (35.5)	0.375
ARBs, n (%)	11 (29.7)	10 (22.2)	0.301
Diuretics, n (%)	13 (35.1)	12 (26.6)	0.278
B-block, n (%)	19 (51.3)	13 (28.8)	0.032
CCA, n (%)	11 (29.7)	19 (42.2)	0.174
Statins, n (%)	11 (29.7)	25 (55.5)	0.016
Fibrates, n (%)	0 (0)	0 (0)	1.000
Ezetimibe, n (%)	0 (0)	3 (6.6)	0.160
Proton pump inhibitors, n (%)	17 (45.9)	18 (40)	0.375
ASA, n (%)	37 (100)	45 (100)	1.000

Data are median (25th–75th percentile).

*BMI* body mass index, *BP* blood pressure, *CVD* cardiovascular disease, *MI* myocardial infarction, *TIA* transient ischemic attack, *PAD* peripheral artery disease, *ACE-I* ACE-inhibitors, *ARBs* angiotensin receptor blockers, *B-block* beta-blockers, *CCA* calcium channel antagonists, *ASA* acetylsalicylic acid.

^a^Determined by Mann–Whitney or x^2^ test, as appropriate.

Patients were comparable for age (p = 0.417), gender (p = 0.448), BMI (p = 0.079), prevalence of hypertension (p = 0.500), CVD (p = 0.407) and C-reactive protein (p = 0.404).

Patients with T2DM were on statin treatment more frequently than patients without T2DM (p = 0.016), while levels of total cholesterol (p = 0.213), HDL (p = 0.588), and triglycerides (p = 0.386) were comparable.

As expected, patients were significantly different for fasting plasma glucose (p < 0.001), HbA1c (p < 0.001) and diabetes specific treatment such as metformin (p < 0.001) and PPAR-gamma agonists (p = 0.012). Notably, median HbA1c in T2DM patients was 6.8% (51.0 mmol/mol), reflecting good glycemic control in the group of patients with diabetes included in the study.

### Repeatability of the outcome measurement

To verify the intra-subject reproducibility of the main outcome measured, we re-assessed EPC number after 24 h in 9 out of 82 patients analyzed. We found no significant difference (p = 0.573) between the two consecutive days measurements (data not shown).

### Effect of T2DM and statin therapy

**Figure 1:**
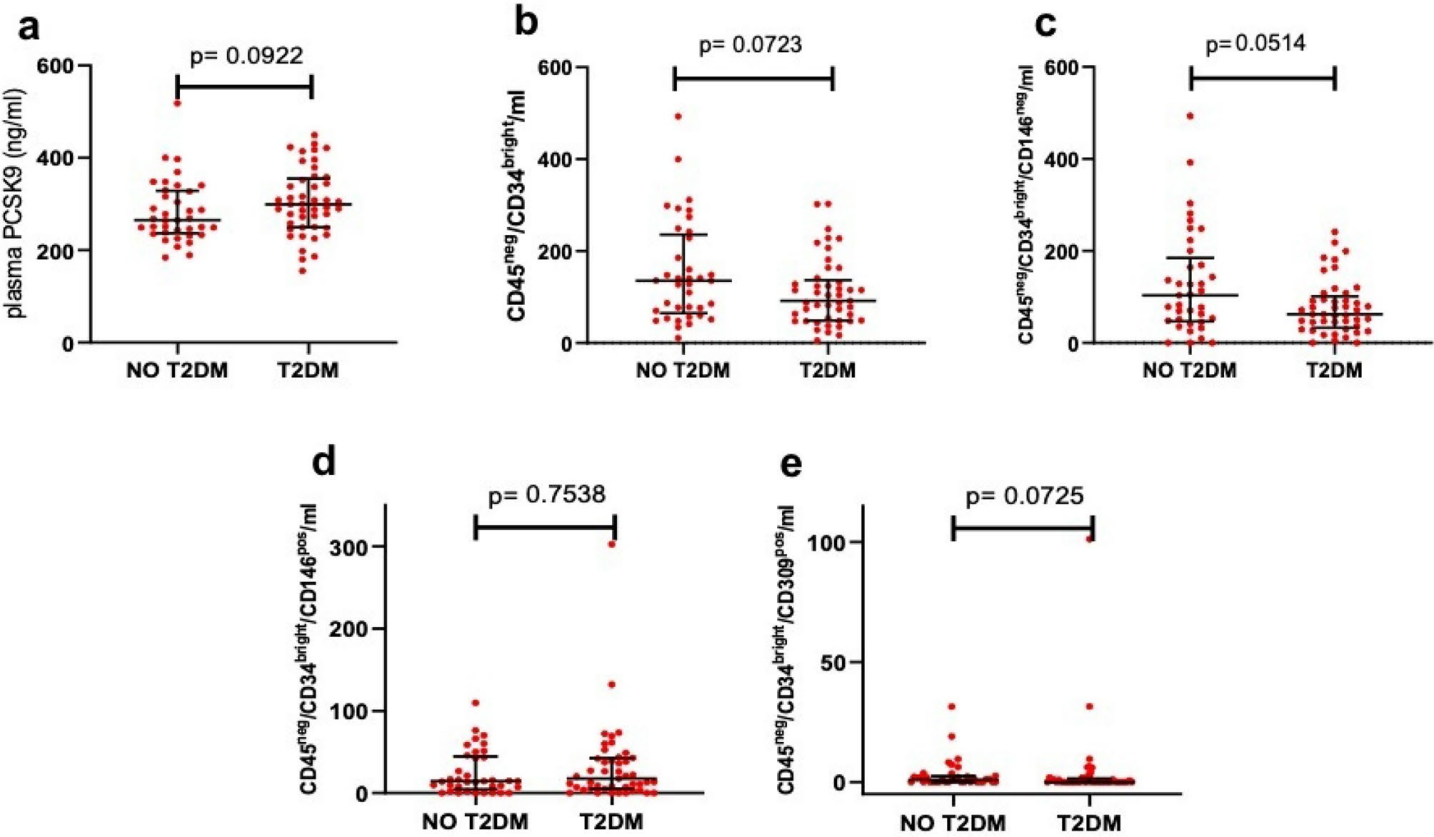
Effect of T2DM. Levels of plasma PCSK9 (**a**) and number of CD45^neg^/CD34^bright^ (**b**), CD45^neg^/CD34^bright^/CD146^neg^ (**c**), CD45^neg^/CD34^bright^/CD146^pos^ (**d**) and CD45^neg^/CD34^bright^/CD309^pos^ (**e**) in patients with and without T2DM.

In patients with T2DM, as compared with patients without T2DM, we observed a trend toward higher levels of plasma PCSK9 (p = 0.092) and lower number of CD45^neg^/CD34^bright^ (p = 0.072), CD45^neg^/CD34^bright^/CD146^neg^ (p = 0.051) and CD45^neg^/CD34^bright^/CD309^pos^ (p = 0.072) although these trends did not achieve statistical significance. The number of CD45^neg^/CD34^bright^/CD146^pos^ was comparable (p = 0.754) (Fig. 1).

**Figure 2:**
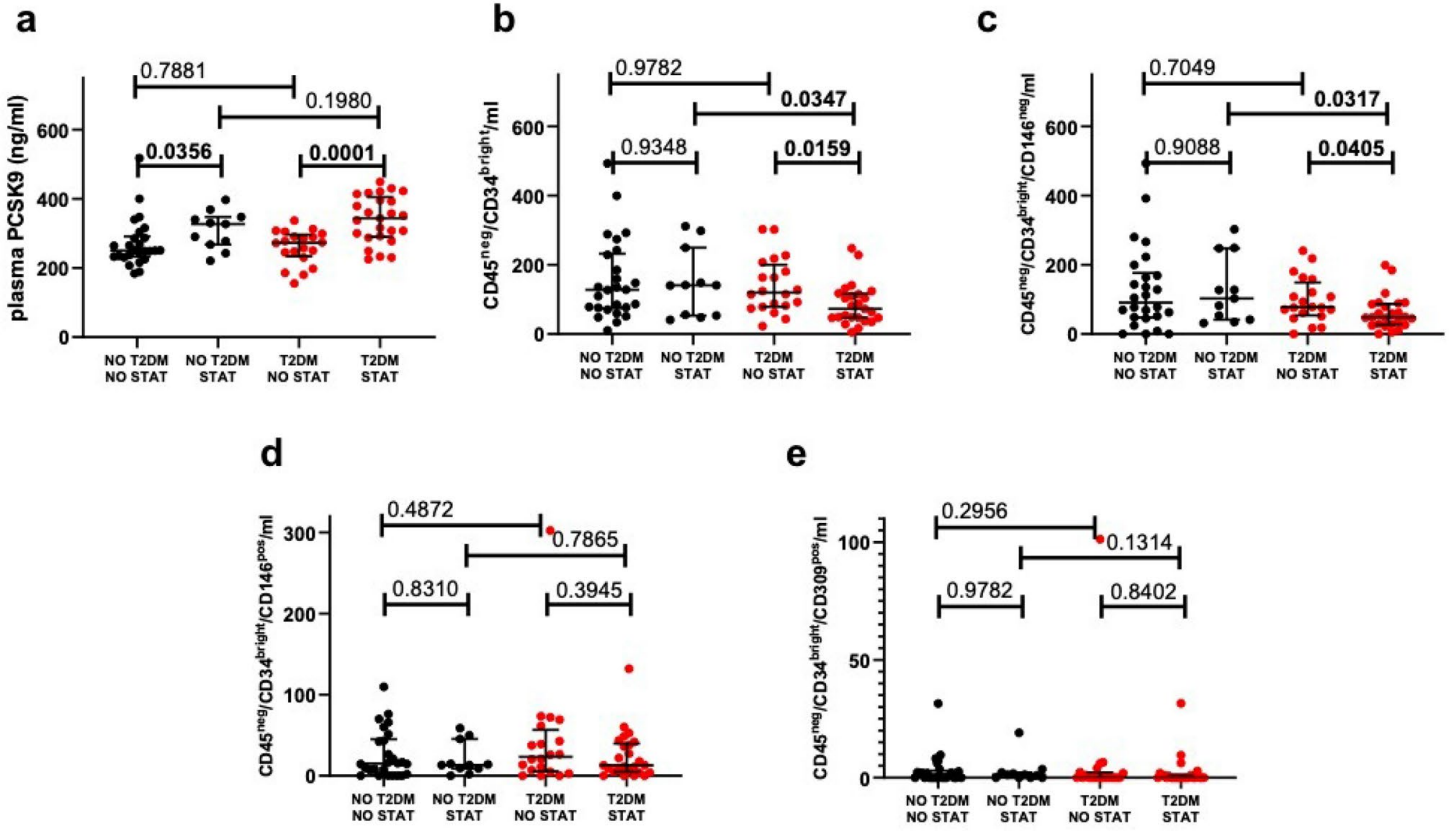
Effect of statin treatment*.* Levels of plasma PCSK9 (**a**) and number of CD45^neg^/CD34^bright^ (**b**), CD45^neg^/CD34^bright^/CD146^neg^ (**c**), CD45^neg^/CD34^bright^/CD146^pos^ (**d**) and CD45^neg^/CD34^bright^/CD309^pos^ (**e**) in statin-treated patients (STAT) and in patients not on statins (NO STAT) with and without T2DM.

Thirty-six patients were on statin treatment, 25 with and 11 without T2DM. Patients on statin treatment had a higher prevalence of diabetes, previous myocardial infarction (MI), lower cholesterol and higher transaminase levels. Otherwise, their clinical characteristics were superimposable. Patients on statin treatment, both with and without T2DM, showed higher levels of PCSK9 as compared to their counterparts not on statins (p < 0.001 and p = 0.036 respectively) (Fig. 2A). Of note, the association of statins with PCSK9 was independent of type of statin used (P = 0.86 for difference among types), of statin dose (P = 0.10 for difference between high versus moderate/low intensity) and of achievement of LDL target (< 70 mg/dL, according to latest ESC guidelines) (P < 0.0001 for the association of statins with PCSK9 after adjustment for LDL target reached (yes *versus* no)). Conversely, CD45^neg^/CD34^bright^ and CD45^neg^/CD34^bright^/CD146^neg^ were significantly lower in statin-treated patients than in patients not on statins, only among patients with T2DM (p = 0.016 and p = 0.040 respectively) (Fig. 2B,C). CD45^neg^/CD34^bright^/CD146^pos^ and CD45^neg^/CD34^bright^/CD309^pos^ were not affected by statin-treatment either in patients with or without T2DM (Fig. 2D,E).

To assess whether cell counts may be further related to statin dose or intensity, we stratified patients accordingly. Namely, seven patients were on high-intensity statins, and 29 on moderate/low-intensity statins. We were not able to assess any relationship between statin dose or intensity and the level of any of the EPC phenotypes analysed. Among individuals with statins, CD45^neg^/CD34^bright^ (p = 0.072), CD45^neg^/CD34^bright^/CD146^neg^ (p = 0.051) and CD45^neg^/CD34^bright^/CD309^pos^ levels were not associated neither with statins type (P = 0.51, P = 0.84, and P = 0.43, respectively) nor with dose of statin (P = 0.22, P = 0.29, P = 0.58) and for difference between high versus moderate/low-intensity, respectively.

Interestingly, among subjects not on statin therapy, no difference was detected in either PCSK9 levels or EPCs number between patients with *vs.* those without T2DM (Fig. 2)**,** while significantly reduced CD45^neg^/CD34^bright^ (p = 0.035) and CD45^neg^/CD34^bright^/CD146^neg^ (p = 0.032) were observed in patients with diabetes *vs*. patients without diabetes only among statin-treated subjects (Fig. 2B,C).

Only among patients not on statins, we observed a positive correlation between PCSK9 and total cholesterol (Rho = 0.369, p = 0.012) and LDL cholesterol (Rho = 0.470 p = 0.001) (Supplementary Fig. 1) but not with HDL or triglycerides (data not shown). Neither PCSK9 nor EPC number were related to fasting plasma glucose or HbA1c (data not shown).

### PCSK9 and EPCs

**Figure 3:**
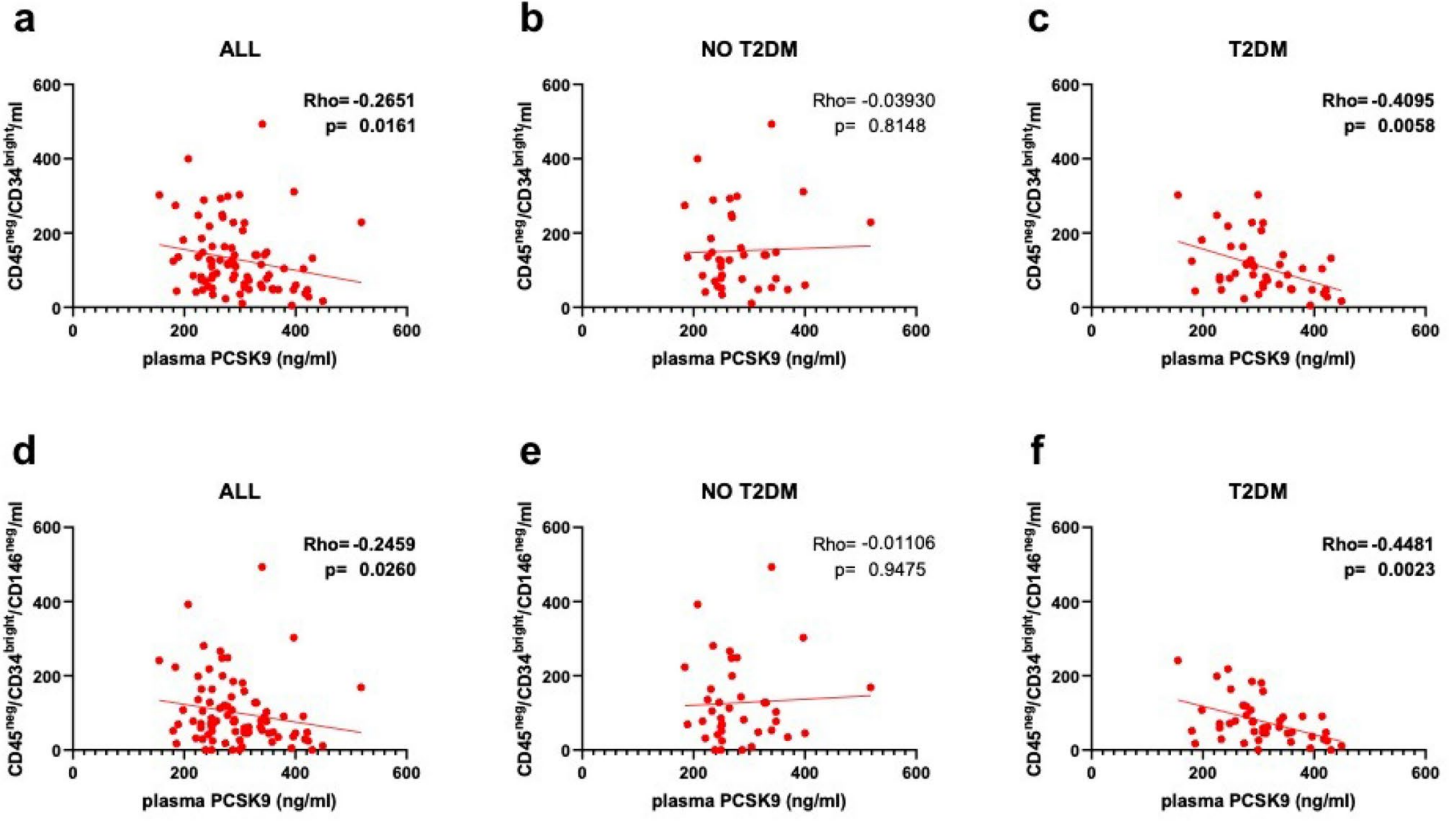
Plasma PCSK9 and EPCs. Correlations between levels of plasma PCSK9 and number of CD45^neg^/CD34^bright^ (top) and CD45^neg^/CD34^bright^/CD146^neg^ (bottom) in patients considered as a whole (**a**,**d**) and in those without (**b**,**e**) and with T2DM (**c**,**f**).

In the whole group, PCSK9 correlated inversely with both CD45^neg^/CD34^bright^ (Rho = − 0.265, p = 0.016) and CD45^neg^/CD34^bright^/CD146^neg^ (Rho = − 0.246, p = 0.026) (Fig. 3A,D). In patients with T2DM, PCSK9 correlated inversely with both CD45^neg^/CD34^bright^ (Rho = − 0.409, p = 0.006) and CD45^neg^/CD34^bright^/CD146^neg^ (Rho = − 0.448, p = 0.002) (Fig. 3C,F), while such correlation was not observed in patients without T2DM (Fig. 3B,E). None of the other EPC phenotypes explored appeared to be correlated with PCSK9 circulating levels (Supplementary Fig. 2).

**Figure 4:**
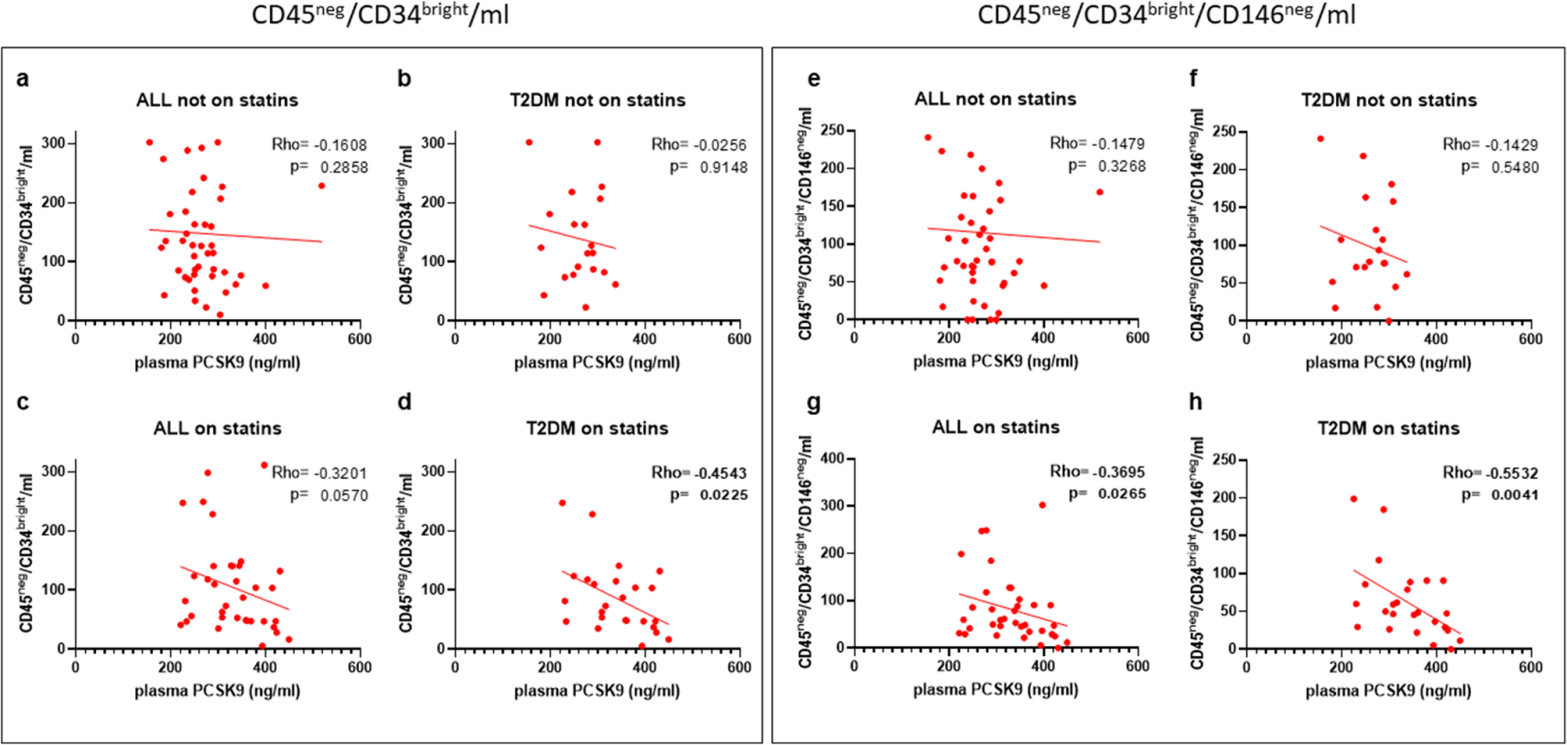
Plasma PCSK9, EPCs and statin treatment*.* Correlations between levels of plasma PCSK9 and number of CD45^neg^/CD34^bright^ (**a**–**d**) and CD45^neg^/CD34^bright^/CD146^neg^ (**e**–**h**) in patients considered as a whole not on statins (**a**,**e**) and on statins (**c**,**g**) and in T2DM patients not on statins (**b**,**f**) and on statins (**d**,**h**).

Since we have observed that statins affect both PCSK9 levels and CD45^ne^g/CD34^bright^ and CD45^neg^/CD34^bright^/CD146^neg^, we made a sub-analysis as a function of statin treatment. Interestingly, when dividing patients according to statin treatment, we found that the inverse correlations between PCSK9 levels and CD45^neg^/CD34^bright^ and CD45^neg^/CD34^bright^/146^neg^ remained only in patients with diabetes on statin treatment (Rho = − 0.454, p = 0.022 and Rho = − 0.553, p = 0.004 respectively) (Fig. 4).

### Effect of previous myocardial infarction (MI)

Interestingly, patients with a previous MI (n = 12) showed significantly higher levels of plasma PCSK9 (p = 0.007), and significantly lower number of CD45^neg^/CD34^bright^/CD146^neg^ (p = 0.031). Again, in patients with a previous MI, the prevalence of ongoing statin treatment was higher than in subjects without a history of previous MI (75% *vs*. 38%, p = 0.025). The other phenotypes were comparable among patients with and without a previous MI (Supplementary Fig. 3).

### Multivariate analyses

Multiple regression analyses performed on all patients, with different EPC phenotypes as the dependent variables, showed that statin treatment was the only independent predictor of the number of CD45^neg^/CD34^bright^ (β = − 0.230; t-value = − 2.113; p = 0.038) (adjusted R^2^ = 0.041, p = 0.038). Multiple regression analyses on T2DM patients showed that PCSK9 circulating levels predicted both the number of CD45^neg^/CD34^bright^ (β = − 0.438; t-value, − 3.199; p = 0.003) (adjusted R^2^ = 0.173, p = 0.003), and CD45^neg^/CD34^bright^/CD146^neg^ (β = − 0.458; t-value, − 3.376; p = 0.002) (adjusted R^2^ = 0.191, p = 0.002) independently of age, gender, BMI and statin treatment.

## Discussion

In this cross-sectional study, we evaluated and compared the circulating levels of PCSK9, the number of several EPC phenotypes and their mutual relationship between patients with T2DM and without T2DM, otherwise comparable for most of the clinical characteristics. The main findings of our study are: 1. Statin treatment is associated with higher circulating levels of PCSK9 in both groups, and with reduced early EPC number only among patients with T2DM; 2. PCSK9 and EPC number are comparable between patients with *vs.* without T2DM, when considering subjects not on statins. In contrast, among statin-treated patients, both the whole CD45^neg^/CD34^bright^ subset as well as the early EPC compartment are reduced in patients with T2DM *vs.* patients without T2DM. 3. Only in the group of T2DM patients, higher circulating PCSK9 is associated with reduced number of early EPCs and CD45^neg^/CD34^bright^ cells. This inverse correlation is not observed in patients without T2DM or in the subgroup of patients not on statins, suggesting that in subjects with diabetes and in particular in statin-treated subjects, enhanced circulating PCSK9 may impair early EPC number, reflecting cardiovascular homeostasis and regenerative capacity. Of note, CEC (CD45^neg^/CD34^bright^/CD146pos) levels were not related to statin therapy, despite acknowledged pleiotropic effects of statins on vascular integrity^29^.

In our patients we observed higher levels of PCSK9 in patients treated with statins, both with and without T2DM. This is in line with the observation that statins can directly upregulate the expression of PCSK9^30,31^. The observed trend toward higher circulating PCSK9 levels in T2DM patients is likely to be driven by the higher prevalence of statin treatment among T2DM (56% *vs*. 30%), rather than by an influence of glucose metabolism on PCSK9. The relationship between PCSK9 and glucose homeostasis is complex and controversial in the literature^32^. Mendelian randomization studies have shown that PCSK9 genetic variants associated with lower LDL cholesterol are also associated with circulating higher fasting glucose concentration and an increased risk of Type 2 diabetes^33^. In experimental mice models, PCSK9 deficiency increased LDLR expression and cholesterol esters accumulation in pancreatic islets, which impairs insulin secretion^34^, although this is likely a local effect, rather than a consequence of circulating PCSK9. The potential impact of PCSK9 on insulin secretion was confirmed in a human biobank, where a PCSK9 46L variant was associated with beta cell dysfunction but not with insulin resistance (HOMA-IR)^35^. Vice versa, insulin is known to induce PCSK9 expression^36^. Recently, a novel crosstalk signal between glucose and cholesterol homeostasis via ChREBP-mediated PCSK9 regulation has been described^37^.

In our study, lack of correlation between fasting plasma glucose or HbA1c and PCSK9 does not support this hypothesis, at least in our cohort.

Additionally, only in patients not in treatment with statins, we found a direct correlation between PCSK9 levels and total cholesterol and LDL. It is known that statin therapy increasing PCSK9 levels and reducing cholesterol levels disrupt their direct correlation^38^.

The effect of statins on EPCs is not well characterized, due to considerable heterogeneity in patient population, cardiovascular risk profile, statin regimens and markers used for EPCs characterization in the diverse studies. Several studies reported a significant increase in EPCs following statin regimen^29,39,40^while others reported a decrease^41^ or no change^42^. Even within patients with chronic coronary artery disease (CAD), divergent results were observed, with significantly increased^39^ or reduced^41^ EPCs counts observed in statin-treated patients vs. matched controls in stable CAD, and an increase^40^ or no alterations despite high-dose statins^42^ after PCI. Inconsistency may be attributed to the diverse phenotypical characterization of EPC, or to dose and duration of statin treatment. Consistent with our findings, early EPCs were found to be significantly higher in patients not treated with statins, whereas late EPCs were significantly higher in statin-treated patients^43^. In addition, long-term statin therapy maintained late EPCs in circulation which may promote neovasculogenesis^44^.

Length of statin treatment seems to be an additional variable: in stable CAD patients, increased circulating EPC were observed as soon as 1 week since first statin intake, with plateauing after 3–4 week^39^; in a prospective analysis, initiation of statin therapy significantly diminished the number of EPCs after 3 but not after 1 month^41^, suggesting EPC impairment with chronic statin use. Consistently, a short-term statin discontinuation increases EPCs in T2DM patients^45^. The observation that statin treatment is associated with reduced number of CD45^neg^/CD34^bright^ and CD45^neg^/CD34^bright^/146^neg^ only among patients with T2DM, suggests that an interaction exists between diabetes and statins. Our study comprised chronic statin users (at least three months), although we cannot exclude suboptimal adherence. Finally, the effect of statins on EPC seems to be dose-dependent, since statin reloading in moderate statin users was associated with increased EPC levels^40,46^.

Interestingly, among the 12 patients with previous MI, PCSK9 and early EPC (CD45^neg^/CD34^bright^/146^neg^) are higher and lower, respectively, as compared to patients without previous MI. Again, these differences may be largely attributed to the higher prevalence of statin treatment among those with a history of previous MI (75% *vs.* 38%). However, our results are in line with previous observations of Laugsand reporting a 47% higher MI risk in patients in the highest quartile of circulating PCSK9^47^. Hill et al. reported a negative correlation between EPCs, measured by colony forming units, and Framingham risk score in 45 men with various degrees of cardiovascular risk. They also reported a positive correlation between CFU and brachial flow-mediated dilation, a measure of endothelial function^48^ Consistently, a meta-analysis of 35 randomized controlled trials found that therapy with PCSK9 inhibitors was associated with a lower rate of MI (2.3% versus 3.6%; odds ratio [OR]: 0.72 [95% confidence interval (CI), 0.64–0.81]; p < 0.001)^49^. Likewise, early EPCs reduction in patients with a previous MI may be a biomarker of endothelial damage and reflect early EPC exhaustion, since early EPC can migrate to the foci of ischemia to promote the repair of the injured organs^50^. In our cohort, we did not observe any differences in CD45^neg^/CD34^bright^ in patients with *vs* without a previous MI. However, this phenotype includes both EPCs and CECs, the latter characterized as CD45^neg^/CD34^bright^/CD146^pos^^23^. Indeed, after excluding the CECs, the remaining CD45^neg^/CD34^bright^/146^neg^ cells, reflecting early outgrowth EPCs^23^, are depleted in patients with a previous MI. Again, it is conceivable that an alteration of early/late EPC balance exists in relation to cardiovascular burden.

Fewer early EPC colonies and higher late EPC colonies were produced in patients with CAD than in control subjects without CAD^51^. The interpretation of the above-mentioned results may have been biased by lack of adjustment for ongoing treatment potentially affecting EPC, including statins.

We hypothesized that EPC impairment may be mediated, at least in part, by PCSK9. Notably, in patients with T2DM, considered as a whole and in those treated with statins, we found an inverse correlation between PCSK9 levels and both CD45^neg^/CD34^bright^ and CD45^neg^/CD34^bright^/146^neg^ phenotypes. The link between PCSK9 and EPCs has been previously observed. In a study published by Chao et al. in patients with peripheral artery disease, high plasma levels of PCSK9 were associated with dysfunction in EPCs^52^. However, in the same study this observation was not paralleled by a reduction in the number of EPCs, at least using the CD34^pos^/KDR^pos^ phenotype. A direct effect of PCSK9 levels on Sca-1/VEGF-R2 EPCs has been observed in a mouse model in which the administration of anti-PCSK9 antibodies increased the number of circulating EPCs^9^, although this cells have be recently recharacterized as B2 lymphocyte upon deeper analysis^53^. Our results add further information to expand this hypothesis in humans. Statin treatment further increases PCSK9. Notably, EPCs impairment is prominent in patients with the highest levels of PCSK9, namely T2DM patients on statin treatment. Whether PCSK9′s influence on EPC is dependent on underlying metabolic abnormality is still uncharacterized. It is conceivable that PCSK9 may impair EPC by modifying insulin secretion and metabolic control, or vice versa that the metabolic derangement of diabetes may alter PCSK9 levels, in turn affecting EPC number. Whichever the underlying mechanisms, our findings of a putative selective effect of PCSK9 on EPC number only in patients with diabetes may provide a mechanistic explanation for the results of the ODYSSEY OUTCOMES trial, a randomised, double-blind, placebo-controlled trial performed in patients on high-intensity statin-treatment, showing that the anti-PCSK9 antibody alirocumab produced about twice the absolute reduction in CV events among patients with diabetes as in those without diabetes^54^. Within diabetes pharmacotherapy, a number of drugs besides statins are know to influence EPC counts^55^. ACE inhibitors have been shown to stimulate EPCs^56^ Ang II potentiates VEGF-induced human EPCs proliferation^56^. Angiotensin II receptor antagonists increase the number of regenerative EPCs in patients with T2DM^57^. Among anti-hyperglycemic agents, rosiglitazone facilitates angiogenic progenitor cell differentiation toward endothelial lineage^58^. The dipeptidyl peptidase-4 (DPP-4) inhibitor sitagliptin increases circulating EPCs in T2DM patients with concurrent upregulation of SDF-1alpha^59^. Treatment with exenatide, but not with liraglutide, is able to increase the number of circulating EPCs, possibly through an antioxidative/antiinflammatory effect^60^. There is no evidence that SGLT2is can directly improve EPC cell levels in T2DM. Thus, cardiovascular protection elicited by SGLT2is should be mediated by other mechanisms^61^. The actual contribution of EPC modulation to the pleiotropic cardioprotective effects of these medications remains unknown^62^. In our study, none of the ongoing medications apart from statins appeared to influence either EPC phenotype.

Limitations include the small sample to test the hypothesis of an interaction between diabetes and statins on early EPC number. However, this pilot study is hypothesis-generating for a previously unappreciated effect of statins, to be confirmed on adequately sized samples. Another limitation is cross-sectional nature of the study. An intervention study assessing the effect of statin treatment on plasma PCSK9 and early EPC number in patients with and without diabetes would have yielded definitive evidence.

Strengths include balance between the groups in terms of clinical characteristics, despite lack of randomization, and the method used to assess EPCs. Indeed, polychromatic flow cytometry used to enumerate and characterize EPCs has a standardized, high sensitive, flexible, and able to quickly analyse thousands of events and multiple parameters at the same time^24,25^.

In conclusion, we unravelled, in patients with T2DM in good glycemic control, already treated with the state-of-the-art strategies for CV prevention (100% on ASA, 55% on statins, 85% antihypertensives), an inverse correlation between circulating PCSK9 and early EPC number, with those on statins showing the highest PCSK9 levels paralleled by the most impaired EPC number. The relatively small sample size and the cross-sectional nature of this study do not allow us to confirm the cause-and-effect relationship between plasma PCSK9 and EPC number, nor a direct influence of statins on this biochemical and cell derangement. However, these findings highlight a piece of the pathophysiology underlying the “residual risk” of high-risk patients optimally treated with current preventive strategies, and suggest that early EPC impairment may be reverted by PCSK9 inhibitors, thus providing an interesting mechanistic explanation for the cardiovascular benefit of this class of drugs and a further indication for the patient with diabetes on top of statins.

## Materials and methods

### Patients recruitment

Forty-five T2DM patients (25 male, median age 68 years), with or without vascular disease, were enrolled at the Diabetes Clinic of Chieti University Hospital. Moreover, we studied 37 patients (22 male, median age 66 years) without T2DM, comparable for demographic, anthropometric and clinical characteristics, with particular reference to cardiovascular risk factors and concurrent treatments, referred to our Clinic by general practitioners. Each subject signed written informed consent to participate, and the Protocol was approved by the Ethics Committee of the University of Chieti (Prot.1129 18.07.2013).

T2DM diagnosis was made according to the ADA criteria (fasting plasma glucose ≥ 126 mg/dL or 2-h plasma glucose ≥ 200 mg/dL during OGTT or HbA1c ≥ 6.5 or a random plasma glucose ≥ 200 mg/dL)^63^. All the patients were in treatment with low-dose aspirin (100 mg/die) for cardiovascular prevention. Exclusion criteria were: uncontrolled hypertension, uncontrolled dyslipidemia, significant comorbidities such as kidney or liver disease, pregnancy or lactation, chronic inflammation, cigarette smoking; clinically significant cardiac and/or pulmonary insufficiency; history of malignant neoplasms (diagnosed and treated within the past 5 years); history of malabsorption; regular (daily) alcohol consumption; regular (i.e., more than 3 days per week) non-steroidal anti-inflammatory drug intake. Type 1 diabetes was excluded by islet autoantibodies evaluation (anti-glutamic acid decarboxylase, islet cell cytoplasmic, and IA-2 antibodies), in the presence of any of the following: family history of type 1 diabetes, age lower than 40 years, lean phenotype, early requirement for insulin therapy. No patient was diagnosed as having MODY (Maturity Onset Diabetes of the Young).

This study was performed under the Good Clinical Practice regulations (Good Clinical Practice for Trial on Medicinal Product-CPMP/European Commission-July 1990; Decreto Ministeriale 27.4.1992-Ministero della Sanità) and the Declaration of Helsinki (Hong Kong 1989). By signing the protocol, the participants in the study committed to adhere to local legal requirements. All methods were performed in accordance with the relevant guidelines and regulations. Informed consent was obtained from all participants and/or their legal guardians.

### PCSK9 levels

Since PCSK9 has a diurnal rhythm^64^, all samples were collected at 8 a.m. after an overnight fasting. Blood collected into EDTA containing vacuum tubes (vacutainer, Becton Dickinson) was centrifuged at 1200×*g* for 10 min at RT to separate plasma. Plasma was aliquoted in small volumes and frozen at − 80 °C. PCSK9 levels were measured with commercial enzyme-linked immunosorbent assays (ELISA) kit (#DPC900, R&D) according to the Manufacturer's instructions. Within-assay and between-assay coefficient of variations were below 7%.

### Circulating endothelial cells

**Figure 5:**
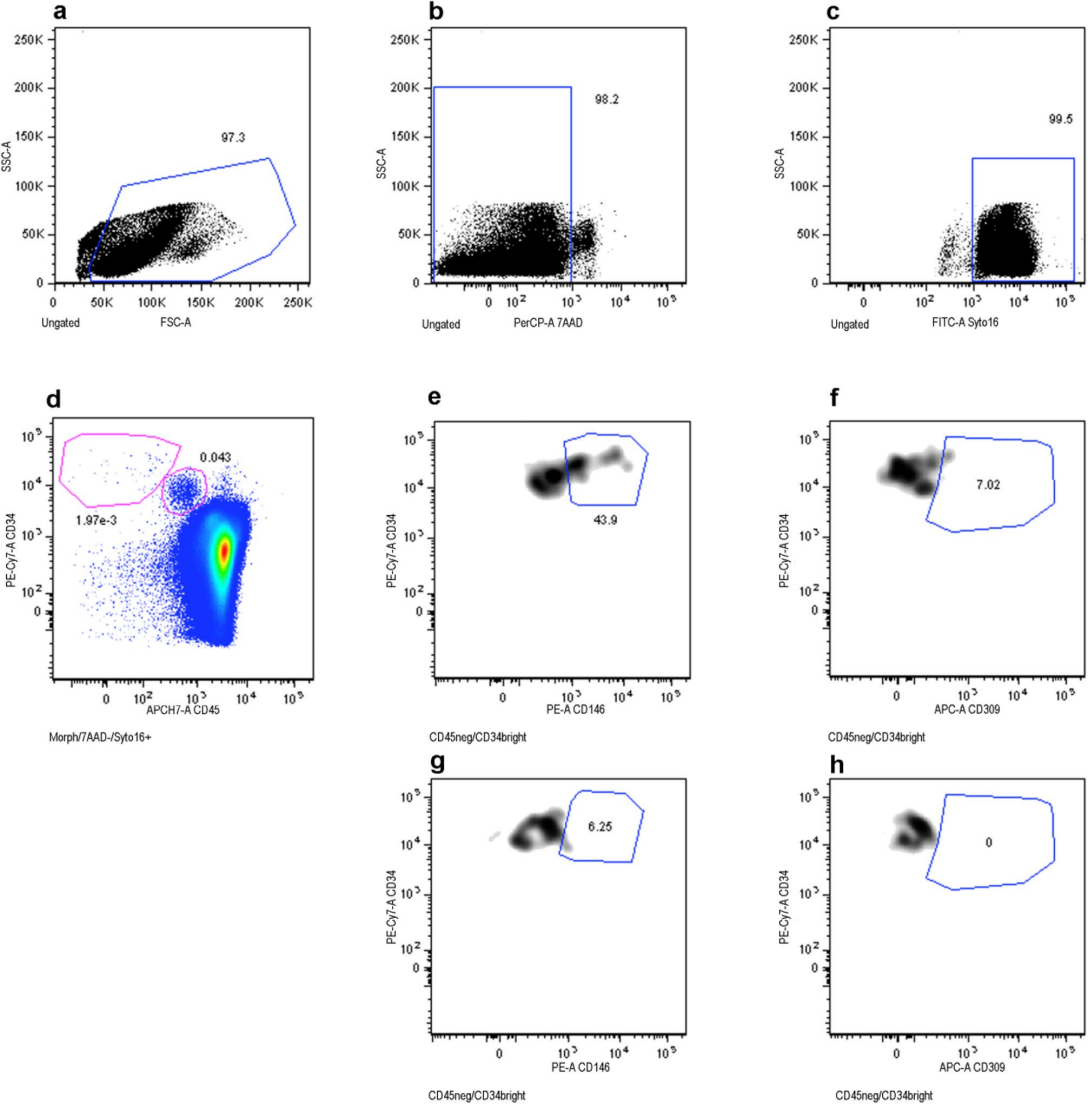
Events displaying the typical lympho-monocyte morphology were first selected in a forward scatter (FSC) versus side scatter (SSC) dot-plot (**a**). Dead cells were excluded on the basis of their positivity to 7-AAD (**b**) and nucleated events (Syto16 + , **c**) were gated. Regions identified in (**a**–**c**) were logically intersected and cells resulting from this combination (lympho-monocyte morphological features, alive and nucleated), were then analysed for their phenotypes. Two subpopulations were identified on a CD45/CD34 dot-plot: CD34 positive cells (hematopoietic stem cells) and a CD34 bright cells (**d**). The CD45^neg^/CD34^bright^ cell population was then analysed for CD146 and CD309 expression, on CD146/CD34 and CD309/CD34 dot plots, respectively (**e**,**f**), and compared with the respective control tube dot-plots, containing the isotype control of the anti-CD146 and anti-CD309 in combination with all the remaining reagents (**g**,**h**).

The analysis of circulating endothelial cells was carried out by plychromatic flow cytometry on peripheral blood samples as already reported^25^. Briefly, 20 × 10^6^ leukocytes/sample underwent an erythrocyte-lysis step (45 mL of Pharm Lyse solution—BD Biosciences—for 15 min at RT, under agitation) and then centrifuged (400*g*, 10 min, room temperature). Once washed (2 mL of Stain Buffer, BD Biosciences), samples were centrifuged and stained using 1 µM Syto16 (Thermo Fisher Scientifc, Eisai, Medipost—US) and a lyophilized cocktail of reagents (BD Biosciences; cat. 623920)^25^. Samples were incubated in the dark for 30 min at 4 °C, washed, centrifuged, re-suspended in 1.5 mL of FACSFlow (BD Biosciences), and finally 2–4 × 10^6^ events/sample with lymph-monocyte were acquired by flow cytometry (FACSCanto, FACSAria, BD Biosciences). A threshold combination was used on Forward Scatter (FSC) and Fluorescein isothiocyanate (FITC-Syto16) channels. Compensations were calculated using CompBeads (BD Biosciences) and single stained fuorescent cells. Carryover between samples was prevented by appropriate instrument cleaning at the end of each sample acquisition. CD45^neg^/CD34^bright^, CD45^neg^/CD34^bright^/CD146^neg^, CD45^neg^/CD34^bright^/CD146^pos^ and CD45^neg^/CD34^bright^/CD309^pos^ phenotypes were analysed (Fig. 5). Briefly, events displaying the typical lymph-monocyte morphology were first selected in a forward scatter (FSC) versus side scatter (SSC) plot (Fig. 5a). Next, dead cells were excluded on the basis of their positivity to 7-AAD (Fig. 5b) and nucleated events (DNApos) were gated (Fig. 5c). The aforementioned three gates were intersected and cells resulting from this logical combination, characterized by lymph-monocyte morphological features, alive and nucleated, were then analysed for their phenotypes. Only non-hematopoietic CD45^neg^ cells were further analysed. The whole CD34positive cell compartment displayed different levels of CD34 surface expression and two subpopulations were identified: CD34 positive cells and a CD34 bright (Fig. 5d). CD45^neg^/CD34^bright^ cell population was then analysed for CD146 and CD309 expression, on CD146/CD34 (Fig. 5e) and CD309/CD34 dot plots (Fig. 5f), respectively, and compared with the respective control tube dot plots, containing the isotype control of the anti-CD146 and anti-CD309 in combination with all the remaining reagents (Fig. 5g,h). The flow cytometry method here described has been previously standardized and published^24,25^. All antibodies and reagents were titrated under assay conditions. The antibody specificity and the gating strategy were defined using fluorescence minus one controls (FMO), as recommended^65^. Subpopulation numbers and abundance were calculated by a dual-platform counting method using the lymphocyte subset as a reference population as reported^24^.

### Statistical analysis

The primary outcome measure is the number of CD45^neg^/CD34^bright^ cells. Our sample size has power = 80% (α = 0.05) to detect a difference greater than 60% of the standard deviation in the primary outcome between patients with diabetes and patients without diabetes, and power = 80% (α = 0.05) to detect an interaction effect between diabetes status and statins use with an effect size > 0.30.

Comparisons of variables between groups were performed by X^2^ tests or Mann–Whitney U tests. Spearman rank correlation test was used to assess relationships among variables. Stepwise multiple linear regression analysis was performed to assess variables independently associated with EPCs. Covariates included in the multiple regression models were selected on the basis of their significance on univariate analysis and their clinical relevance to the outcome of interest. They included diabetes, statin treatment, PCSK9 levels, age and gender, BMI.

Only 2-tailed probabilities were used for testing statistical significance, and p < 0.05 was considered statistically significant. All calculations were carried out using SPSS (SPSS, Chicago, IL, USA). Sample size and power analysis have been conducted by using of GPower^66^.

## Supplementary Information

Supplementary Information
