# Supplementary material for: Endogenous PCSK9 may influence circulating CD45neg/CD34bright and CD45neg/CD34bright/CD146neg cells in patients with type 2 diabetes mellitus
Source: Sci Rep. 2021 May 6;11:9659. doi: 10.1038/s41598-021-88941-x (PMC8102605; doi:10.1038/s41598-021-88941-x)
Supplement: Supplementary file 1 — Supplementary Information [file 41598_2021_88941_MOESM1_ESM.docx]

Endogenous PCSK9 may influence circulating CD45^neg^/CD34^bright^ and CD45^neg^/CD34^bright^/CD146^neg^ cells in patients with type 2 diabetes mellitus

Romina Tripaldi, Paola Lanuti, Paola Giustina Simeone, Rossella Liani, Giuseppina Bologna, Sonia Ciotti, Pasquale Simeone, Augusto Di Castelnuovo, Marco Marchisio, Francesco Cipollone, and Francesca Santilli

**Supplementary Data**

**
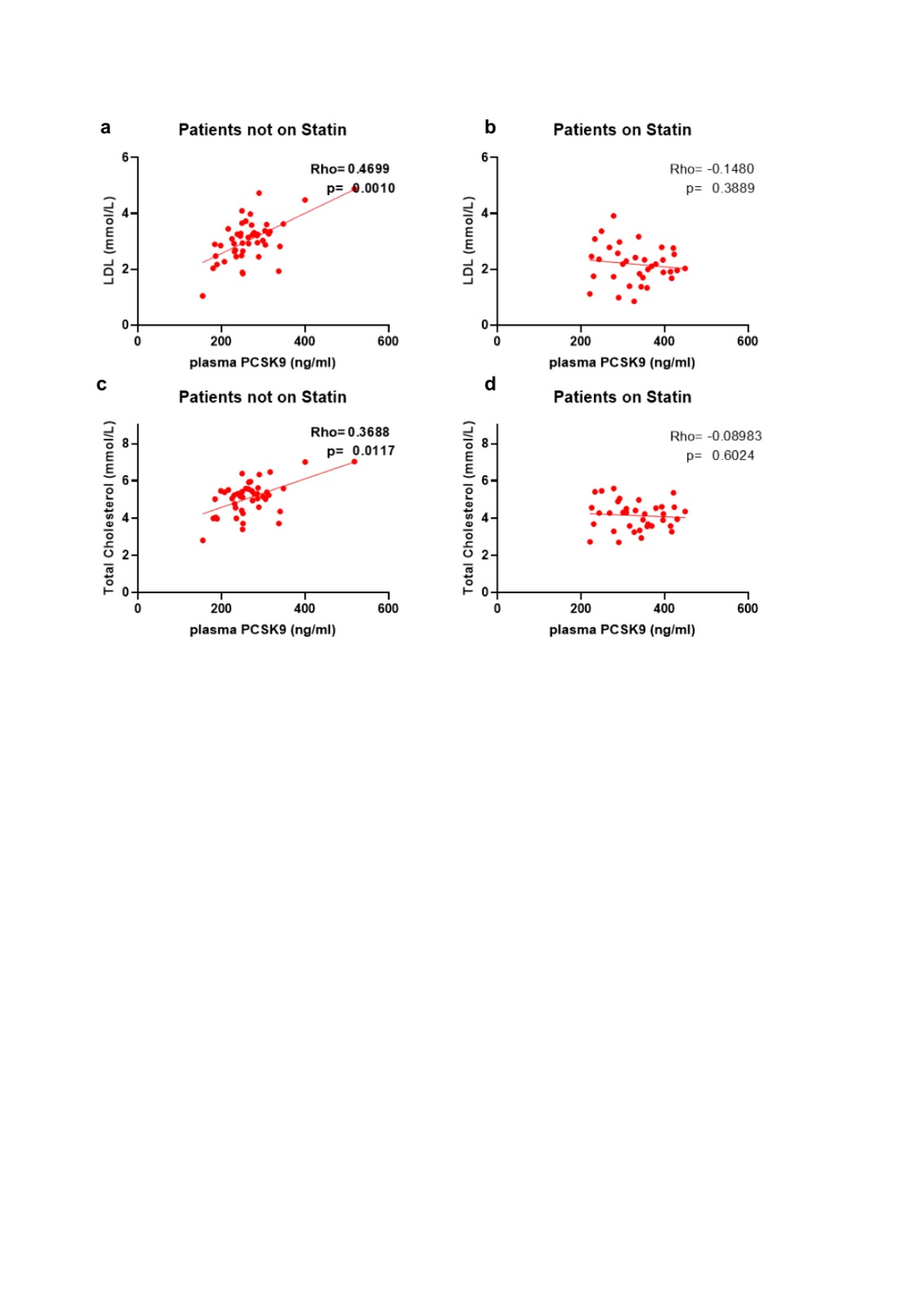
**

**Supplementary Figure 1.** Plasma PCSK9 and cholesterol. Correlations between levels of plasma PCSK9 and LDL cholesterol (a-b) and total cholesterol (c-d) in statin-treated patients and in patients not on statins.

**
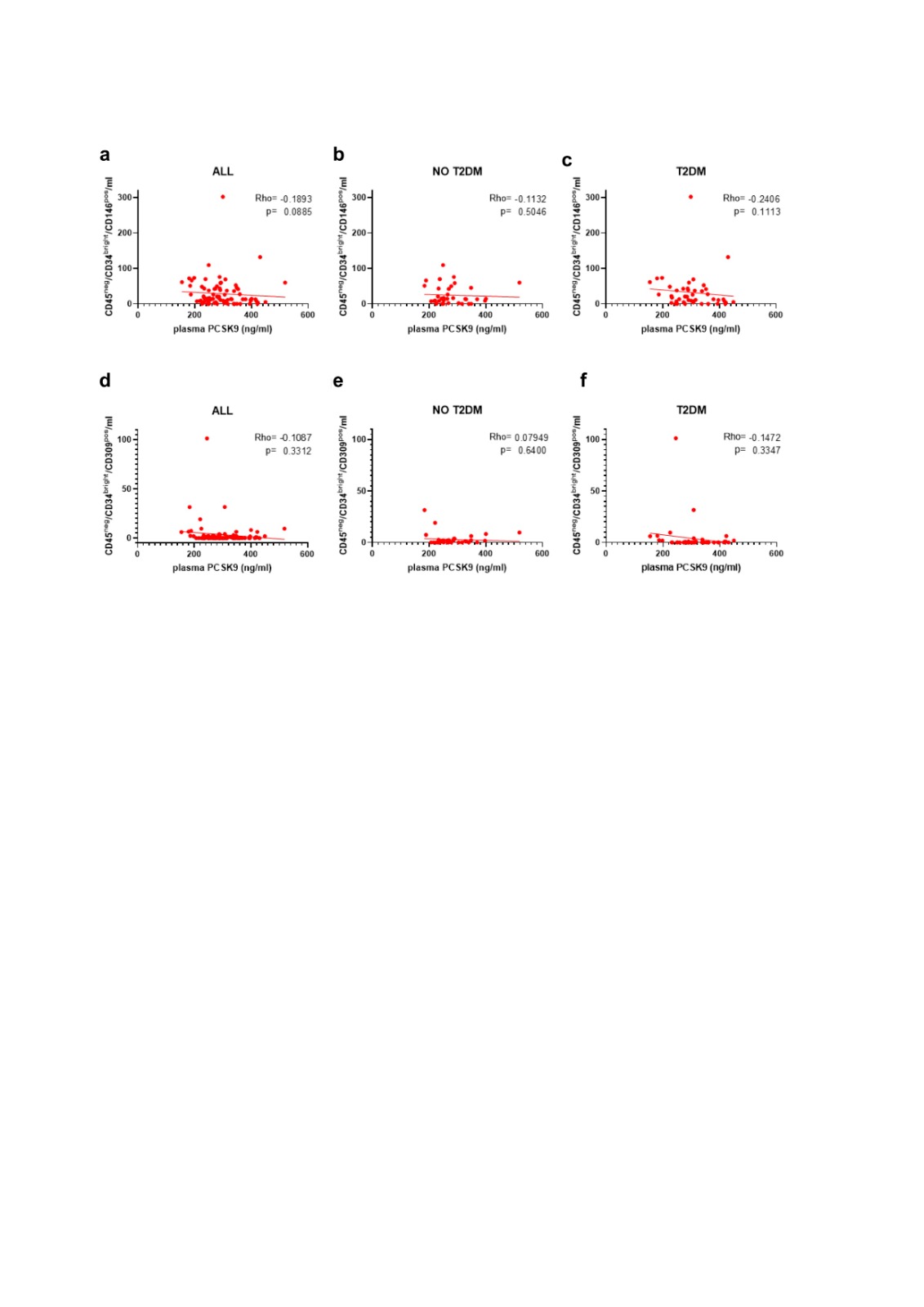
**

**Supplementary Figure 2. Plasma PCSK9 and EPCs.** Correlations between levels of plasma PCSK9 and number of CD45^neg^/CD34^bright^/CD146^pos^ (top) and CD45^neg^/CD34^bright^/CD309^pos^ (bottom) in patients considered as a whole (a,d) and in those without (b,e) and with T2DM (c,f).

**
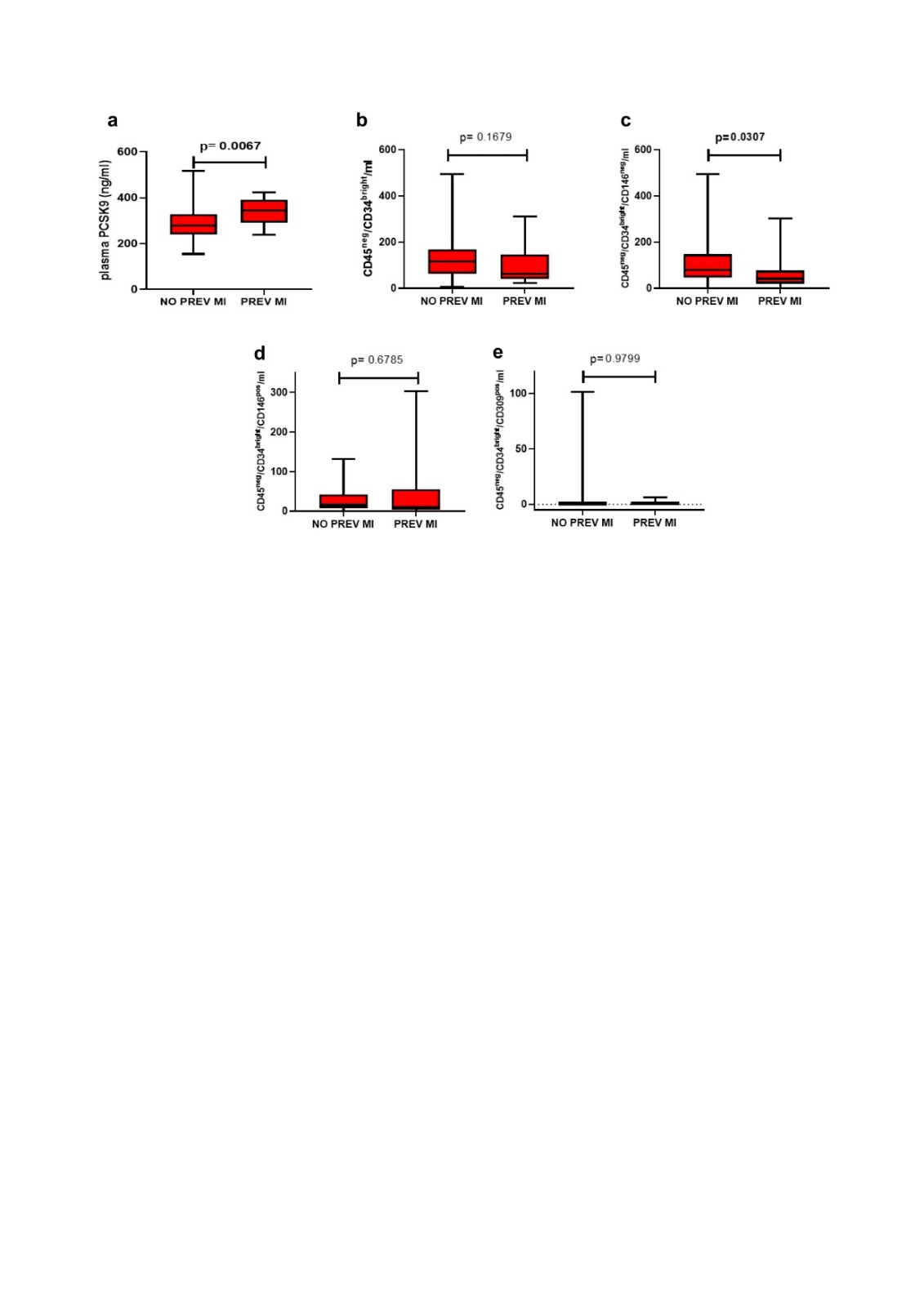
**

**Supplementary Figure 3.** Effect of previous MI. Levels of plasma PCSK9 (a) and number of CD45^neg^/CD34^bright^ (b), CD45^neg^/CD34^bright^/CD146^neg^ (c), CD45^neg^/CD34^bright^/CD146^pos^ (d) and CD45^neg^/CD34^bright^/CD309^pos^ (e) in patients with and without a previous MI.
